# Energy input-output analysis of paddy fields under different irrigation modes and nitrogen fertilizer managements

**DOI:** 10.3389/fpls.2026.1789853

**Published:** 2026-03-25

**Authors:** Yinge Zhang, Ying Liu, Haijun Liu, Yimeng Zhu, Tangzhe Nie, Siyuan Wang, Lili Jiang, Tianyi Wang, Peng Chen, Zhongyuan Guo

**Affiliations:** 1 School of Water Conservancy and Electric Power, Heilongjiang University, Harbin, China; 2 Heilongjiang Provincial Hydrology and Water Resources Center, Harbin, China; 3 College of Agricultural Science and Engineering, Hohai University, Nanjing, China; 4 Qing’an County Northern Oasis Rice Research Institute, Suihua, China

**Keywords:** water-nitrogen coupling, controlled irrigation, flood irrigation, rice cultivation, energy use patterns

## Abstract

Coupled water–nitrogen management has the potential to improve rice productivity while reducing energy demand, yet its integrated effects on the energy balance of cold-region rice production systems remain insufficiently quantified. To address this issue, a two-year field experiment was conducted in Northeast China from 2021 to 2022 to evaluate the interactive effects of irrigation regime and nitrogen (N) rate on rice yield and energy dynamics. The experiment adopted a factorial design with two irrigation modes, flood irrigation (F) and controlled irrigation (C), and four N application rates (0, 85, 110, and 135 kg N ha^-1^), resulting in eight treatments with three replicates each. Energy input (EI), energy output (EO), net energy (NE), and energy use efficiency (EUE) were quantified for all treatments. The results showed that the highest N rate (135 kg N ha^-1^) produced the greatest EI across treatments and increased dependence on non-renewable energy, while diesel, machinery, and fertilizers together accounted for more than 73% of total EI. Under controlled irrigation, C–N110 achieved the highest EO (292,046 MJ ha^-1^) and NE (252,425.25 MJ ha^-1^), and its grain yield was significantly higher than that of the other treatments (P < 0.05), indicating that improved energy performance was accompanied by enhanced productivity. Under flood irrigation, F–N110 reduced EI compared with F–N135 while maintaining comparable EO, suggesting superior energy performance under a stable water supply. Overall, these findings demonstrate that both irrigation regime and N rate strongly affect the energy performance of rice production in cold regions, and that controlled irrigation combined with a moderate N rate of 110 kg ha^-1^ is an effective strategy for improving both rice yield and energy efficiency.

## 1 Introduction

Agricultural production is one of the major sectors of global energy consumption (Erdal et al., 2007). According to the FAO, the global population will reach 9.7 billion by 2050, necessitating a doubling of global agricultural output. The sector accounts for approximately 70% of all freshwater withdrawals worldwide and a substantial share of global energy use (Turetta et al., 2025). However, this “high input for high output” model not only intensifies pressure on non-renewable energy resources–such as those used in diesel fuel and fertilizer production–but also conflicts sharply with the “dual carbon” goals and the imperative of sustainable agricultural development, due to greenhouse gas emissions from energy use, which contribute approximately 10%–12% of global anthropogenic emissions (Singh et al., 2019). Ensuring food security while reducing energy consumption and associated environmental impacts has become a major challenge for sustainable agricultural production. Achieving high crop energy output (EO) under low energy input (EI) conditions has therefore emerged as a key strategy for addressing these challenges.

Among existing crops, rice is consumed by approximately 56% of the global population and serves as a primary food source for 40% of the world’s poor (Verma et al., 2021), playing a critical role in global food security and human nutrition (Fitzgerald et al., 2008). Meanwhile, the energy consumption profile of rice production is distinct compared to that of wheat and maize, rice requires substantial additional EI for irrigation, accounting for 25%–40% of total energy use, and for nitrogen fertilizer production, which constitutes over 60% of the total energy consumed in fertilization. However, in current rice production systems, the conventional “high irrigation + high nitrogen” model results in energy use efficiency (EUE) of only 1.2–1.8, well below the 2.0 threshold required for sustainable agriculture. This practice also leads to secondary environmental issues, including nitrogen loss–contributing to water body eutrophication–and inefficient use of irrigation water, with global rice irrigation accounting for 70% of total agricultural water withdrawals.

Regarding the effects of different irrigation modes on rice energy dynamics, there is a consensus in the academic community that controlled irrigation reduce irrigation related energy consumption, such as diesel for pumping and machinery operations, by reducing both the frequency and volume of irrigation application, thereby lowering total EI. Nitrogen application is another factor affecting energy dynamics, Jin et al (Jin et al., 2023). found that under rainfed and dry-wet alternating irrigation modes, EO peaked at a nitrogen rate of 150 kg ha^-^¹. Du et al (Du et al., 2022). reported that rice achieved maximum yield at a nitrogen application amount of 110 kg ha^-^¹ under both controlled and conventional flood irrigation. This discrepancy suggests a coupling effect between irrigation modes and nitrogen fertilizer applications on EO during rice cultivation. However, existing studies have not systematically quantified this interaction. Therefore, investigating this coupling relationship is crucial for accurately assessing the EI and EO associated with rice production.

Energy calculation is a standard approach for assessing the efficiency of production systems, enabling researchers to quantify input-output ratios and other energy-related metrics in agriculture (Ozkan et al., 2004b). For planners and policymakers, input-output analysis serves as a reliable methodology, providing a systematic pathway to evaluate energy use, efficiency and associated economic benefits (Ozkan et al., 2011). Energy assessment offers a quantitative framework for analyzing agricultural sustainability across countries and regions (Jin et al., 2023). Currently, however, the specific mechanisms through which the coupling effect between irrigation and nitrogen application influences the final energy output (EO) and energy use efficiency (EUE) in rice production systems in Northeast China remain poorly understood. In addition, it remains unclear which combinations of irrigation regime and nitrogen management practices lead to optimal net energy (NE), and how these interactions vary across different environmental and management conditions. To address this knowledge gap, a two-year field experiment (2021–2022) was conducted in Northeast China to study the energy coupling between irrigation modes and nitrogen fertilizer management strategies. The study aims to: (1) analyze the effects of different irrigation methods and nitrogen fertilizer management on rice yield, direct and indirect EI, and the proportion of renewable versus non-renewable energy, and (2) assess the impact of irrigation methods and nitrogen fertilizer management on key energy indicators–including EI, EO and EUE to propose evidence-based recommendations for optimizing irrigation and nitrogen fertilizer management in rice production.

## 2 Materials and methods

### 2.1 Survey of test area

**Figure 1:**
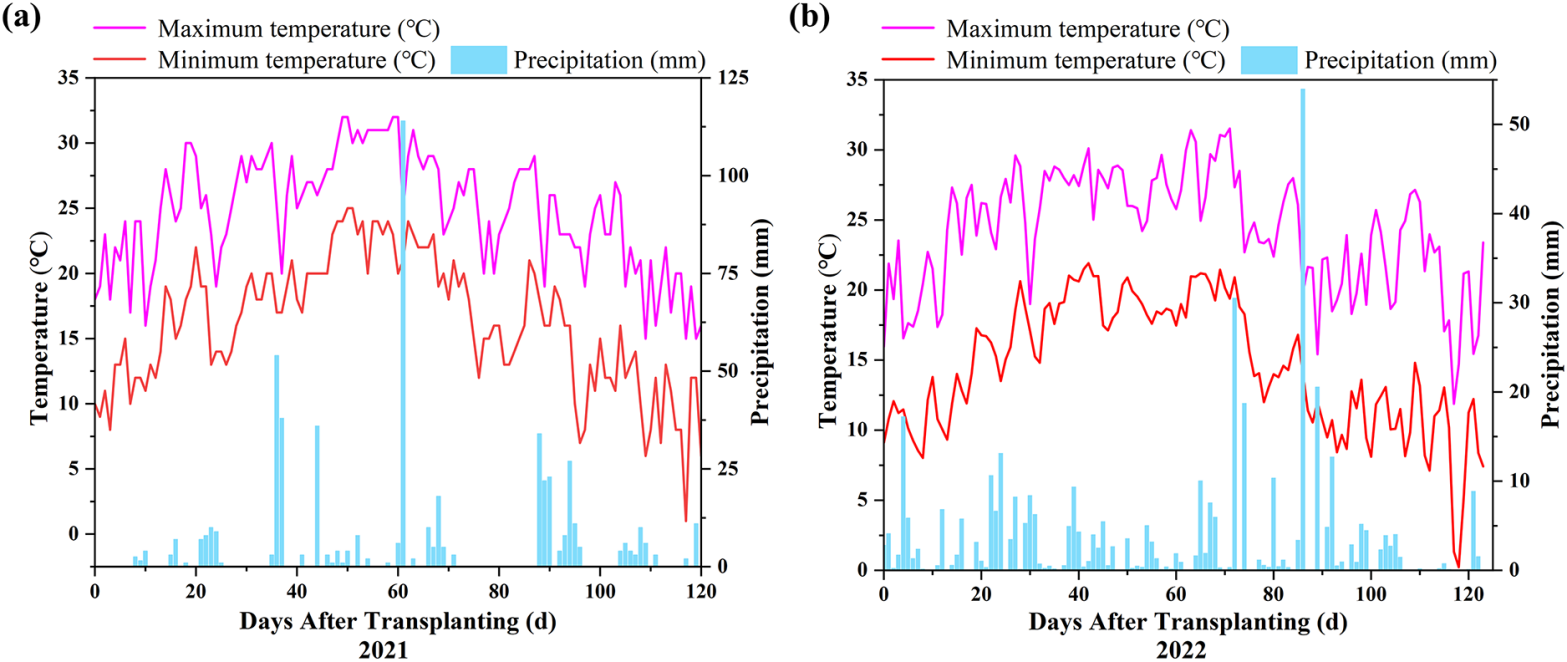
Meteorological data during the rice growing season in **(a)** 2021 and **(b)** 2022.

The experiment was conducted from 2021 to 2022 at the State Key Irrigation Experimental Station, located in Heping Town, Qing’an County, Suihua City, Heilongjiang Province, China (127°40′45″E, 46°57′28″N). This region has a cold temperate continental monsoon climate. The experimental site is characterized by an annual average of 2,599 hours of sunshine, a mean annual temperature of 1.69 °C, a frost-free period of approximately 128 days, and an average annual precipitation of 577 mm. The soil is classified as sandy clay loam, with a pH of 6.5, field capacity of 31.5%, and total porosity of 55.6%. The meteorological data chart for the growth period of rice in 2021 and 2022 is shown in **Figure 1**.

### 2.2 Experimental design

This study was conducted using the widely cultivated local rice variety “Suijing 18”. The planting and harvest dates were May 16 and September 15 in 2021, and May 19 and September 23 in 2022, respectively. Planting density was maintained at 30 cm × 10 cm, with three seedlings per hill. The experimental design was based on two irrigation modes–controlled irrigation (C) and flood irrigation (F)–and four nitrogen application amounts (0, 85, 110, and 135 kg·ha^-^¹, corresponding to N0, N85, N110, and N135, respectively), resulting in 8 treatments. Phosphorus fertilizer (P_2_O_5_, 45 kg·ha^-^¹) and potassium fertilizer (K_2_O, 80 kg·ha^-^¹) were uniformly applied across all treatments, with nitrogen was applied in a ratio of 4.5:2:3.5 (basal fertilizer: tillering stage: panicle stage). Each treatment was replicated 3 times, yielding a total of 24 experimental plots. The plots were separated by concrete ridges, and independently irrigated and drained, each covering an area of 100 m² (10 m × 10 m), and equipped with water meters to measure the volume of irrigation water applied. Soil moisture content was measured at 7:00 a.m. and 6:00 p.m. using the TRIME-PICO64/32 soil moisture analyzer. Irrigation was controlled during the early and midtillering stages and the milk to mature stage, with the lower limit set at 70% of soil saturated soil water content and the upper limit at 100%. During the jointing and flowering stages, the lower irrigation threshold was set at 80% of soil saturated water content, with the upper limit maintained at 100% of soil saturated water content. For the remaining growth periods, the water management regime followed the flooding irrigation practice. Throughout the rice growth period, no standing water layer was maintained, except for a shallow water layer of 0–30 mm during the green-up stage and natural drying during the late maturity phase under controlled irrigation. In the flood irrigation treatment, a water depth of 30–50 mm was maintained continuously throughout the growing season, with the exception of natural drying allowed during the late tillering and maturity stages. Irrigation was initiated when soil moisture content approached or fell below the predetermined lower limit. Irrigation timing and water quota were adjusted according to root- zone moisture conditions. The upper irrigation limit was defined as 100% of saturated soil water content, while the lower limit was set at a specific percentage of this value for each growth stage. This management approach ensured that soil moisture content remained within the target range–between the upper and lower thresholds–for each corresponding growth period. Surface water depth was monitored using a pre-installed vertical ruler (accuracy: 0.1 cm) to determine irrigation requirements. At maturity, rice plants were harvested from a 2 m² area in each plot to assess grain yield.

The rice straw yield was directly measured in our study. At maturity, three representative plants were selected from each treatment, and the above-ground parts of the samples were divided into leaves, stems, sheaths, and panicles, which were then bagged separately. The samples were heated at 105 °C for 30 minutes to halt metabolism, then dried at 85 °C until they reached a constant weight. After cooling, the dry weight of the whole rice plants at different growth stages was measured using an electronic scale with an accuracy of 0.01g. The rice panicles were manually threshed, and the grain dry weight was measured to calculate the yield, which was adjusted to a moisture content of 14%. Finally, the straw yield was determined by subtracting the grain yield from the total dry matter of the rice plants. In the study region, rice straw is typically removed from fields after harvest. To reflect this common practice, straw in each plot was collected and removed after harvest. In the energy accounting, the straw component of EO was calculated as the theoretical gross energy potential of straw biomass (straw yield × Energy equivalent), and thus does not necessarily represent energy that is actually recovered or used in practice.

### 2.3 Energy analysis

This study conducted an energy analysis of eight different rice production system treatments, comparing their differences in EI, EO, and EUE. Inputs at each growth stage, from transplanting to maturity, were identified and quantified. In general, paddy field inputs include machinery, diesel, labor, fertilizers, pesticides, irrigation water, electricity, and seeds, with the energy required for irrigation pumping incorporated into the electricity input. The total dry weight of the mature crop–grains and straw combined–was used as the yield measure. Energy equivalents presented in **Table 1** were derived from published literature and applied to estimate EI and EO during rice production. Agronomic inputs were calculated on a per hectare basis, and the input quantities across treatments are summarized in **Table 2**. Each input was multiplied by its corresponding energy equivalent to compute the EI contribution of that component. Total EI was obtained by summing all individual input energies. EO was calculated by multiplying the grain and straw yields by their respective energy equivalents.

**Table 1:** Energy equivalents of input and output in agricultural production.

Particulars	Unit	Energy equivalent (MJ unit^-1^)	References
A.Inputs			
1.Machinery	h	62.70	(Singh et al., 2008)
2.Labor	h	1.96	(Eskandari and Attar, 2015)
3.Diesel	L	56.31	(Royan et al., 2012)
4.Chemical fertilizer			
Nitrogen(N)	kg	66.14	(Mohammadi et al., 2008)
Phosphorus(P_2_O_5_)	kg	12.44	(Esengun et al., 2007)
Potassium(K_2_O)	kg	11.15	(Yuan and Peng, 2017)
5.Electricity	Kw h	11.93	(Yuan and Peng, 2017)
6.Water	m³	1.02	(Ozkan et al., 2004a)
7.Pesticide			
Herbicide	kg	238.00	(Pathak and Bining, 1985)
Insecticide	kg	199.00	(AghaAlikhani et al., 2013)
Fungicide	kg	216.00	(Pathak and Bining, 1985)
8.Seed	kg	14.70	(AghaAlikhani et al., 2013)
B.Outputs			
1.Rice grain	kg	17.00	(Eskandari and Attar, 2015)
2.Straw	kg	12.50	(Sayin et al., 2005)

**Table 2:** Amounts of energy input in paddy fields under different irrigation systems and nitrogen fertilizer management.

Year	Water management methods	Nitrogen level	Mechanical (h ha^-1^)	Labor force (h ha^-1^)	Diesel (L ha^-1^)	Irrigation water (m³ ha^-1^)	Chemical fertilizer (kg ha^-1^)	Electricity (Kwh ha^-1^)	Pesticide (kg ha^-1^)	Seed (kg ha^-1^)
2021	C	N0	196.0	63.5	210.0	3765.0	125.0	36.0	4.1	18.0
		N85	197.0	64.2	211.0	3765.0	210.0	37.0	4.5	18.0
		N110	198.0	64.9	211.0	3765.0	235.0	38.0	4.6	18.0
		N135	198.0	65.4	213.0	3765.0	260.0	39.0	4.8	18.0
	F	N0	202.0	76.0	220.0	5880.0	125.0	53.0	4.2	18.0
		N85	204.0	76.5	221.0	5880.0	210.0	53.0	4.6	18.0
		N110	204.0	77.3	223.0	5880.0	235.0	54.0	4.7	18.0
		N135	205.0	77.7	223.0	5880.0	260.0	55.0	4.8	18.0
2022	C	N0	198.0	64.2	212.0	4950.0	125.0	49.0	4.3	18.0
		N85	198.0	64.6	213.0	4950.0	210.0	50.0	4.7	18.0
		N110	199.0	65.3	214.0	4950.0	235.0	52.0	4.8	18.0
		N135	199.0	66.5	214.0	4950.0	260.0	52.0	5.0	18.0
	F	N0	204.0	77.1	224.0	7365.0	125.0	71.0	4.3	18.0
		N85	204.0	77.8	224.0	7365.0	210.0	74.0	4.7	18.0
		N110	205.0	78.4	225.0	7365.0	235.0	74.0	5.0	18.0
		N135	206.0	78.9	225.0	7365.0	260.0	75.0	5.2	18.0

Different lowercase letters indicate significant differences among treatments within the same year (p<0.05); Controlled irrigation (C) and flooded irrigation (F). Under each irrigation regime, four nitrogen application rates of 0(N0), 85(N85), 110(N110), and 135(N135) kg N ha^-1^ were applied.

EI for rice production can be categorized into direct and indirect energy. Direct energy includes diesel, irrigation water, electricity, and labor used during crop production. Indirect energy encompasses the embodied energy associated with the manufacture of machinery, fertilizers, pesticides, and seeds. In addition, agricultural energy inputs can be classified into renewable and non-renewable sources based on the nature of the resources. Renewable energy includes irrigation water, human labor, and seeds, whereas non-renewable energy includes machinery, diesel, electricity, fertilizers, and pesticides.

To assess various energy indicators, the calculation formulas for energy input (EI) and energy output (EO), net energy (NE), energy utilization efficiency (EUE), specific energy (SE), energy productivity (EP), and energy profitability (EPF) are given as follows (Demircan et al., 2006; Singh et al., 2019):


Net energy =energy output −Energy input 

$$\text{Energy utilization efficiency }=\frac{\text{energy output }}{\text{Energy input}}$$

$$\text{Specific energy}=\frac{\text{Energy input}}{\text{Grain yield}}$$

$$\text{Energy production efficiency }=\frac{\text{Grain yield}}{\text{Energy input}}$$

$$\text{Energy profitability }=\frac{\text{Net energy}}{\text{Energy input}}$$

### 2.4 Data analysis

Differences in various energy indicators of rice under different irrigation modes and nitrogen fertilizer management were analyzed using two-factor analysis of variance (ANOVA) at a significance level of p< 0.05. The main effects of irrigation and nitrogen fertilizer management were evaluated through two-way ANOVA. Both analyses were conducted using IBM SPSS Statistics 27. All figures were generated using Origin 2021 (OriginLab Corporation, USA), while tables were prepared and visualized using Microsoft Excel 2019 (Microsoft Corporation, USA).

## 3 Results and analysis

### 3.1 Energy input for rice production

EI for rice under different irrigation modes and nitrogen fertilizer management is presented in **Table 3**. Under controlled irrigation, N135 exhibited the highest total EI at 40,619.55 MJ·ha^-^¹ (2021) and 43,993.62 MJ·ha^-^¹ (2022), while N0 had the lowest total EI at 31,191.38 MJ·ha^-^¹ (2021) and 32,841.51 MJ·ha^-^¹ (2022). Under flood irrigation, N135 also showed the highest total EI at 42,151.45 MJ·ha^-^¹ (2021) and 46,018.91 MJ·ha^-^¹ (2022), whereas N0 recorded the lowest values at 34,538.87 MJ·ha^-^¹ (2021) and 36,644.25 MJ·ha^-^¹ (2022). Across all treatments, F-N135 had the highest total EI, while C-N0 had the lowest. Over the two-year period, energy consumption for rice under controlled irrigation ranged from 31,191.38 to 40,619.55 MJ·ha^-^¹, compared to 34,538.87 to 46,018.91 MJ·ha^-^¹ under flood irrigation.

**Table 3:** EI of rice under different irrigation systems and nitrogen fertilizer management.

Year	Water management methods	Nitrogen level (MJ ha^-1^)	Mechanical (MJ ha^-1^)	Labor force (MJ ha^-1^)	Diesel (MJ ha^-1^)	Irrigation water (MJ ha^-1^)	Chemical fertilizer (MJ ha^-1^)	Electricity (MJ ha^-1^)	Pesticide (MJ ha^-1^)	Seed (MJ ha^-1^)	Total energy input (MJ ha^-1^)
2021	C	N0	12289.2	124.5	11825.1	3840.3	1451.8	429.5	966.4	264.6	31191.4
		N85	12351.9	125.8	11881.4	3840.3	7073.7	441.4	1060.9	264.6	37040.1
		N110	12414.6	127.2	11881.4	3840.3	8727.2	453.3	1084.5	264.6	38793.2
		N135	12414.6	128.2	11994.0	3840.3	10380.7	465.3	1131.9	264.6	40619.6
	F	N0	12665.4	149.0	12388.2	5997.6	1451.8	632.3	990.0	264.6	34538.9
		N85	12790.8	150.0	12444.5	5997.6	7073.7	632.3	1084.5	264.6	40438.0
		N110	12790.8	151.5	12557.1	5997.6	8727.2	644.2	1108.1	264.6	42241.2
		N135	12853.5	152.3	12557.1	5997.6	10380.7	656.2	1131.6	264.6	43993.6
2022	C	N0	12414.6	125.8	11937.7	5049.0	1451.8	584.6	1013.4	264.6	32841.5
		N85	12414.6	126.6	11994.0	5049.0	7073.7	596.5	1107.9	264.6	38626.9
		N110	12477.3	128.0	12050.3	5049.0	8727.2	620.4	1131.5	264.6	40448.3
		N135	12477.3	130.3	12050.3	5049.0	10380.7	620.4	1178.8	264.6	42151.5
	F	N0	12790.8	151.1	12613.4	7512.3	1451.8	847.0	1013.2	264.6	36644.3
		N85	12790.8	152.5	12613.4	7512.3	7073.7	882.8	1107.9	264.6	42398.0
		N110	12853.5	153.7	12669.8	7512.3	8727.2	882.8	1178.8	264.6	44242.7
		N135	12916.2	154.6	12669.8	7512.3	10380.7	894.8	1226.0	264.6	46018.9

Different lowercase letters indicate significant differences among treatments within the same year (p<0.05); Controlled irrigation (C) and flooded irrigation (F). Under each irrigation regime, four nitrogen application rates of 0(N0), 85(N85), 110(N110), and 135(N135) kg N ha^-1^ were applied.

As nitrogen fertilizer application increased, the total EI of rice also increased (**Table 3**). Under a nitrogen amount of 135 kg·ha^-^¹, energy consumption ranged from approximately 40,619.55 to 43,993.62 MJ·ha^-^¹ in 2021 and from 42,151.45 to 46,018.91 MJ·ha^-^¹ in 2022, which was substantially higher than that under N0. Nitrogen amounts of 85 and 110 kg·ha^-^¹ resulted in intermediate EI levels. With increasing nitrogen fertilizer use, inputs of pesticides, labor, diesel, and machinery in rice production also increased (**Table 2** and **3**). Concurrently, the proportion of EI from fertilizers to total EI increased with higher nitrogen application. When no nitrogen fertilizer was applied, the total EI of rice was the lowest compared to treatments with 85, 110, and 135 kg·ha^-^¹ nitrogen (**Table 3**), ranging from approximately 31,191.38 to 34,538.87 MJ·ha^-^¹ in 2021 and 32,841.51 to 36,644.25 MJ·ha^-^¹ in 2022. At the same nitrogen level, controlled irrigation resulted in lower total EI than flood irrigation, suggesting that reducing irrigation amount and pumping requirements (electricity/diesel) is a key pathway for lowering EI.

**Figure 2:**
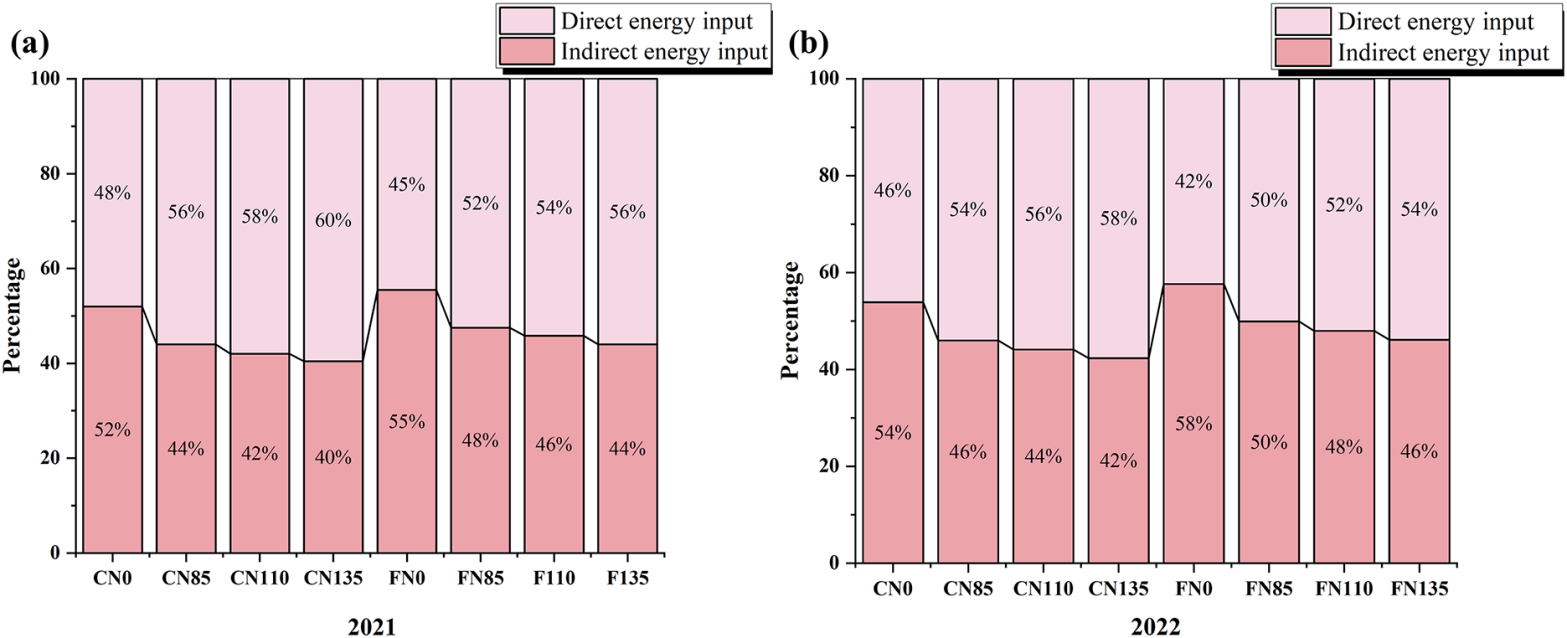
Proportion of direct and indirect energy input in rice production under different irrigation modes and nitrogen fertilizer managements in **(a)** 2021 and **(b)** 2022. Different lowercase letters indicate significant differences among treatments within the same year (p<0.05); Controlled irrigation (C) and flooded irrigation (F). Under each irrigation regime, four nitrogen application rates of 0(N0), 85(N85), 110(N110), and 135(N135) kg N ha^-1^ were applied. (n=3).

**Figure 3:**
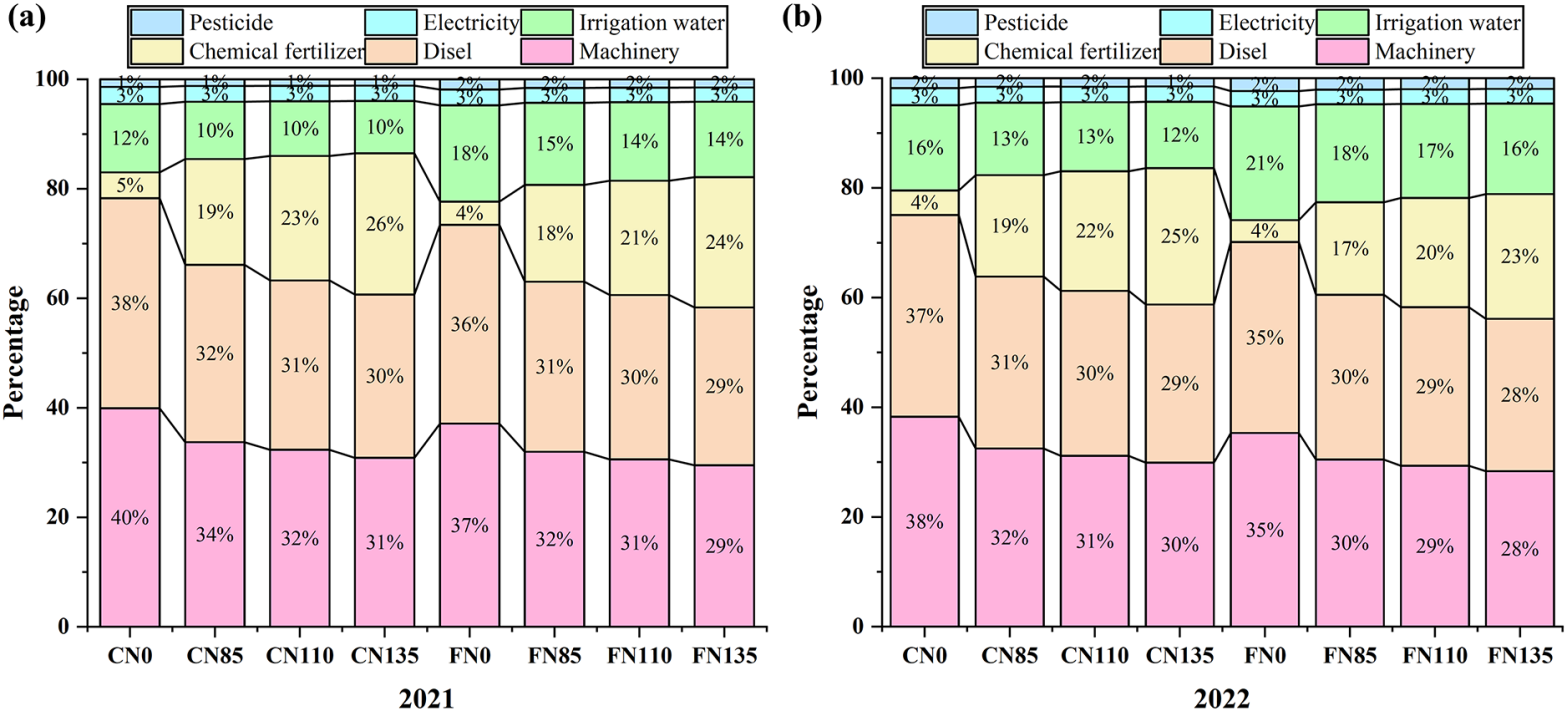
Proportion of energy input to total energy input in rice production under different water and nitrogen management measures in **(a)** 2021 and **(b)** 2022. Different lowercase letters indicate significant differences among treatments within the same year (p<0.05); Controlled irrigation (C) and flooded irrigation (F). Under each irrigation regime, four nitrogen application rates of 0(N0), 85(N85), 110(N110), and 135(N135) kg N ha^-1^ were applied. (n=3).

As shown in **Figure 2**, direct EI of rice accounted for approximately 42% to 60% of the total EI over the two-year period. Under the same irrigation mode, the proportion of direct EI in total EI increased with increasing nitrogen fertilizer application. According to the two-year experimental data (**Figure 3**), diesel contributed the largest share of direct energy, accounting for 28% to 38% of total EI, with the highest proportion observed under the C-N0 (38%). Irrigation water was the second-largest contributor, providing about 4% to 26% of total EI, peaking at 26% under the C-N135. Mechanical operations represented the largest component of indirect EI contributing 28% to 40% of total EI, indicating that mechanized operations and irrigation pumping are the key energy hotspots. Under the same irrigation regime, increasing nitrogen rates raised the proportion of direct EI and also increased indirect EI through the embodied energy of N fertilizer. Overall, water-saving irrigation can reduce irrigation-related direct EI, whereas moderate N application helps prevent concurrent increases in indirect toggle together energy demand and operational energy consumption.

**Figure 4:**
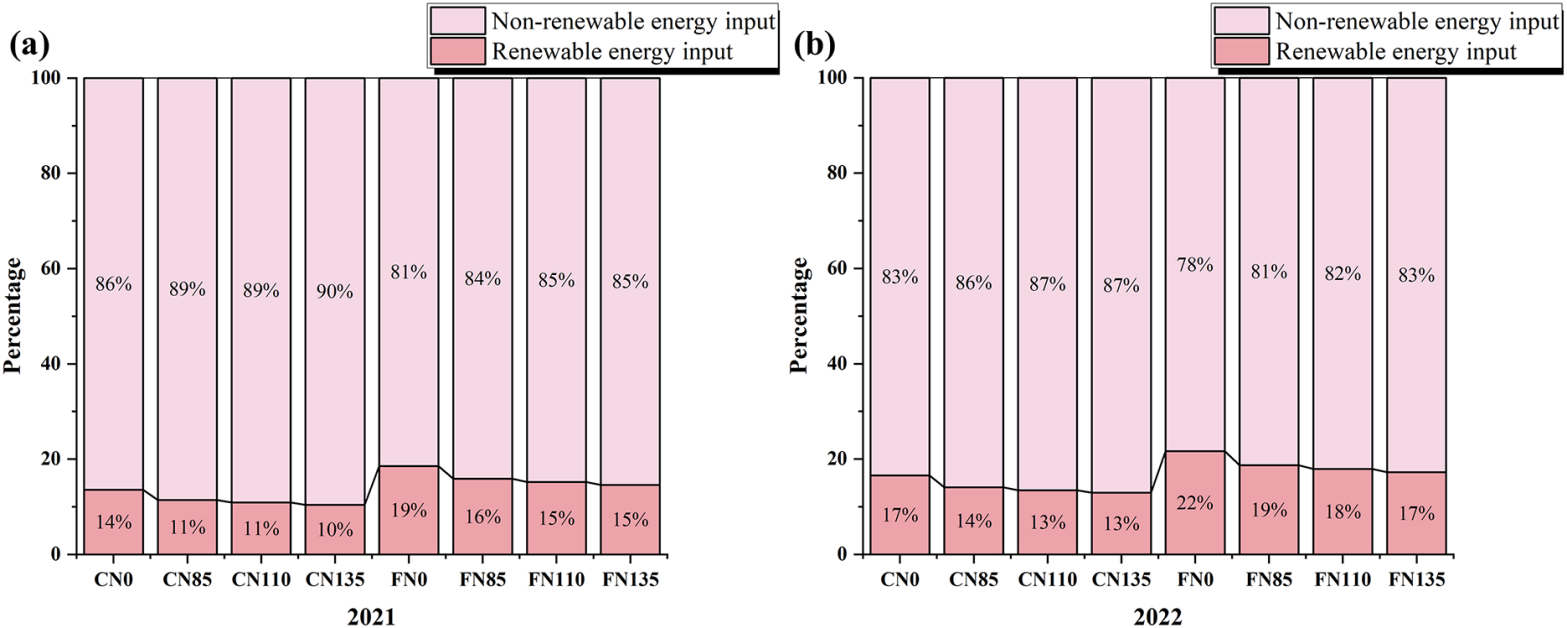
Proportion of renewable and non-renewable energy input in rice production under different water and nitrogen management measures in **(a)** 2021 and **(b)** 2022. Different lowercase letters indicate significant differences among treatments within the same year (p<0.05); Controlled irrigation (C) and flooded irrigation (F). Under each irrigation regime, four nitrogen application rates of 0(N0), 85(N85), 110(N110), and 135(N135) kg N ha^-1^ were applied. (n=3).

As shown in **Figure 4**, renewable EI for rice accounted for approximately 10% to 22% of the total EI over the two-year period. Under the same irrigation mode, the proportion of renewable EI relative to total EI was lowest under N135 and highest under N0. The contribution of non-renewable EI across all production stages was substantially greater than that of renewable EI. Overall, these findings demonstrate a structural dependence of the rice production system on non-renewable energy, higher nitrogen rates increase fertilizer-related embodied energy and associated operational energy (fuel/electricity), thereby enlarging the non-renewable EI component and reducing the relative share of renewable EI. This explains why moderate nitrogen rates combined with water-saving irrigation are more favorable for improving the energy structure and lowering total EI.

### 3.2 Energy output of rice production

**Figure 5:**
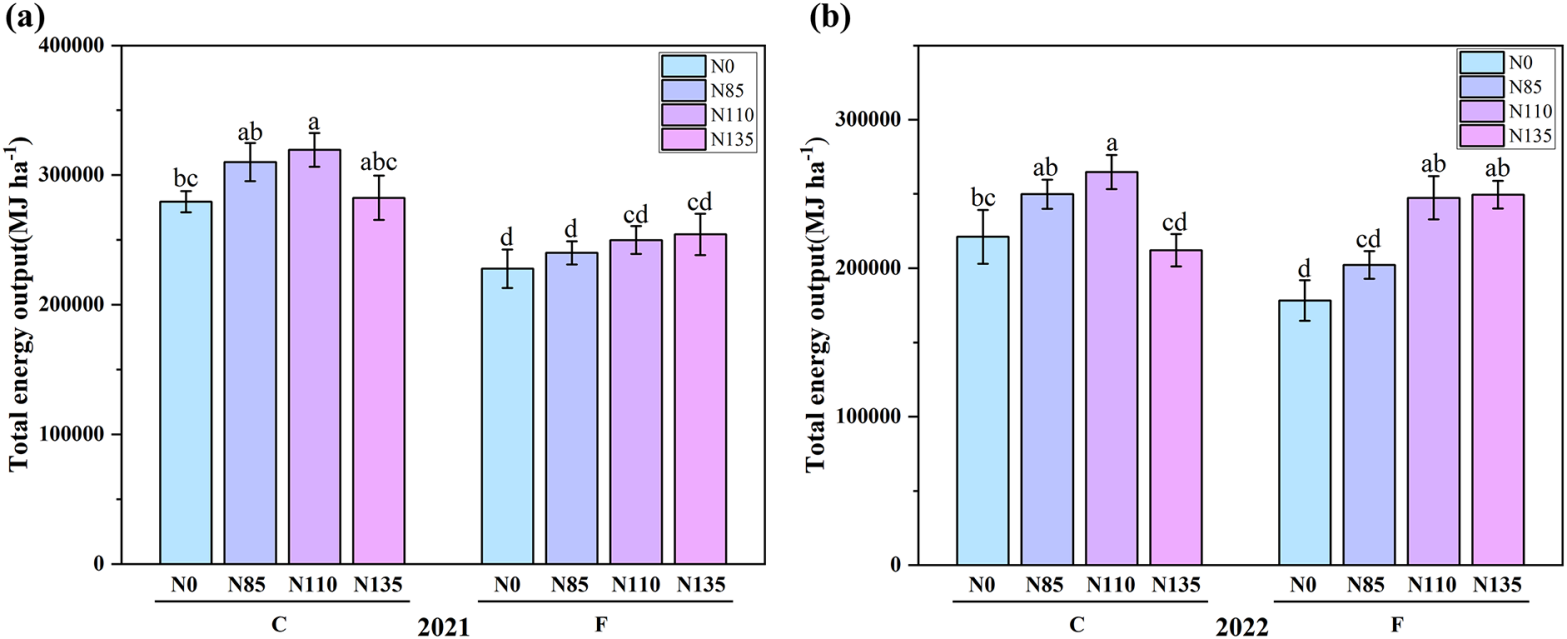
Total energy output of rice under different water and nitrogen management methods in **(a)** 2021 and **(b)** 2022. Error bars indicate the standard errors of the means (n=3); Different lowercase letters indicate significant differences among treatments within the same year (p<0.05); Controlled irrigation (C) and flooded irrigation (F). Under each irrigation regime, four nitrogen application rates of 0(N0), 85(N85), 110(N110), and 135(N135) kg N ha^-1^ were applied.

**Figure 6:**
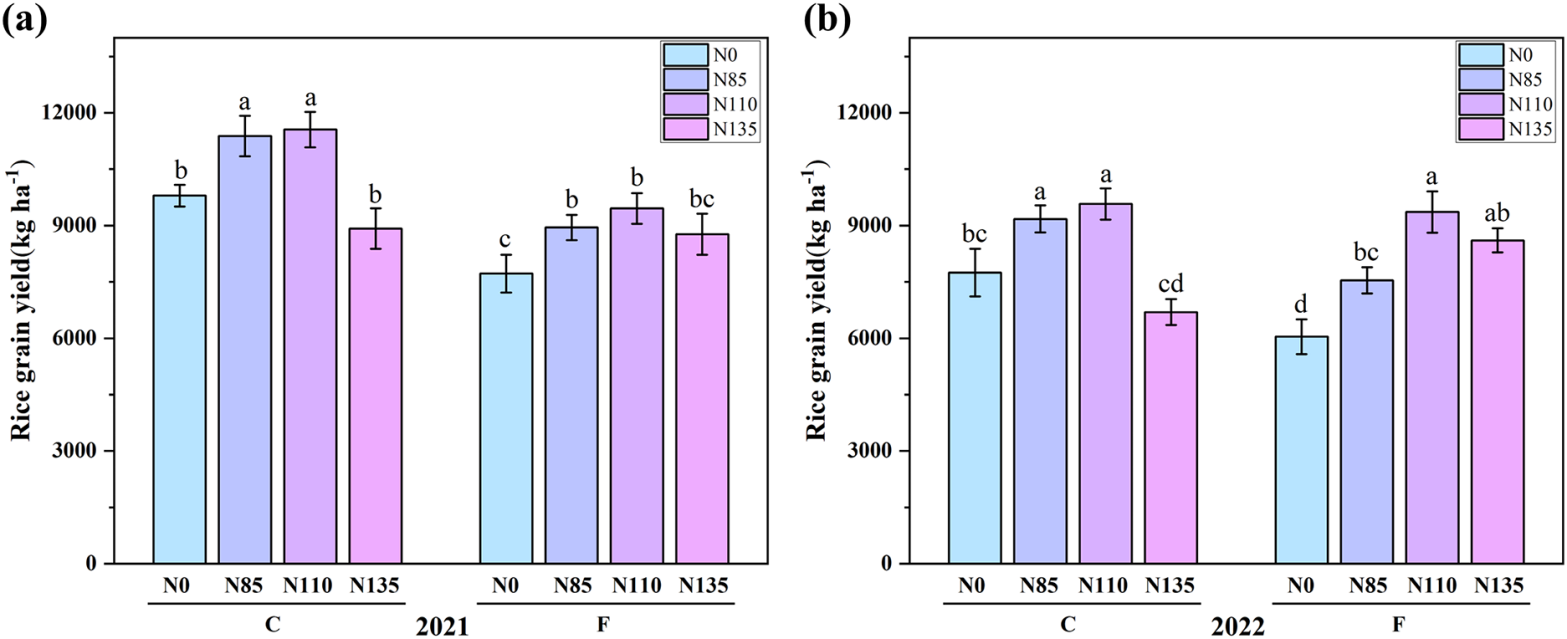
Rice grain yield under different water and nitrogen management methods in **(a)** 2021 and **(b)** 2022. Error bars indicate the standard errors of the means (n=3); Different lowercase letters indicate significant differences among treatments within the same year (p<0.05); Controlled irrigation (C) and flooded irrigation (F). Under each irrigation regime, four nitrogen application rates of 0(N0), 85(N85), 110(N110), and 135(N135) kg N ha^-1^ were applied.

As shown in **Figure 5**, under controlled irrigation, rice total EO exhibited an increasing-then-decreasing trend with rising nitrogen amounts, peaking at C-N110 with 319,421.8 MJ·ha^-^¹ in 2021 and 264,657.7 MJ·ha^-^¹ in 2022. Under flooding irrigation, total EO increased with nitrogen application amount, reaching the highest value under F-N135 at 254,267.5 MJ·ha^-^¹ in 2021 and 249,546.5 MJ·ha^-^¹ in 2022. However, at the same nitrogen fertilizer amount, controlled irrigation resulted in higher EO than flooding irrigation, likely because it improves root-zone conditions and water–nutrient coordination, while excessive N leads to diminishing returns. The lowest EO across the two-year experiment was observed under F-N0, with values of 227,799 MJ·ha^-^¹ in 2021 and 178,209.5 MJ·ha^-^¹ in 2022. F-N135 achieved the highest EO among all flooding irrigation treatments. In **Figure 6**, the N110 treatment has the highest yield in both the 2021 and 2022, the higher yield in the controlled irrigation in 2021 could be due to optimal weather conditions and the increased availability of nitrogen during early crop growth. In 2022, however, the crop may have faced unfavorable conditions or different growth dynamics, resulting in lower overall yields, with N110 showing better responses.

### 3.3 Analysis of energy indicators for rice production

**Table 4** and **5** present the analysis of variance for indicators NE, EUE, SE, EP and EPF of paddy fields under different irrigation modes and nitrogen application amounts. Both irrigation mode and nitrogen fertilizer application amount had significant effects (p ≤ 0.01) on NE, EUE, SE, EP, and EPF, while the interaction between the two factors also significantly influenced these five energy indicators (p ≤ 0.01).

**Table 4:** Analysis of energy indicators of paddy fields under different irrigation modes and nitrogen fertilizer managements in 2021.

2021						
Water management methods	Nitrogen level	Net energy (NE)	Energy utilization efficiency(EUE)	Specific energy(SE)	Energy production efficiency(EP)	Energy profitability(EPF)
C	N0	248318.66ab	8.96a	0.19b	5.34a	7.96a
	N85	272866.67ab	8.36a	0.19b	5.22a	7.36a
	N110	280816.36a	8.27a	0.20b	5.09a	7.27a
	N135	241815.28b	6.95b	0.27a	3.73b	5.95b
F	N0	193260.13c	6.60bc	0.26a	3.80b	5.60bc
	N85	199586.70c	5.94c	0.27a	3.76b	4.94c
	N110	207652.17c	5.92c	0.26a	3.81b	4.92c
	N135	210278.05c	5.78c	0.30a	3.39b	4.78c
W		**	**	**	**	**
N		*	**	**	**	**
W×N		*	*	*	**	*

Error bars indicate the standard errors of the means (n=3);Different lowercase letters indicate significant differences among treatments within the same year (p<0.05); Controlled irrigation (C) and flooded irrigation (F). Under each irrigation regime, four nitrogen application rates of 0(N0), 85(N85), 110(N110), and 135(N135) kg N ha^-1^ were applied. * indicates an interaction at the 0.05 level (p ≤ 0.05), ** indicates an interaction at the 0.01 level (p ≤ 0.01).

**Table 5:** Analysis of energy indicators of paddy fields under different irrigation modes and nitrogen fertilizer managements in 2022.

2022						
Water management methods	Nitrogen level	Net energy (NE)	Energy utilization efficiency(EUE)	Specific energy(SE)	Energy production efficiency(EP)	Energy profitability(EPF)
C	N0	188274.50bc	6.73a	0.27d	3.71a	5.73a
	N85	211156.07ab	6.47a	0.30cd	3.30abc	5.47a
	N110	224209.41a	6.54a	0.29d	3.43ab	5.54a
	N135	169909.39cd	5.03b	0.32bcd	3.17abcd	4.03b
F	N0	141565.25d	4.86b	0.36bc	2.80cde	3.86b
	N85	159802.87cd	4.77b	0.42a	2.37e	3.77b
	N110	203143.99ab	5.59b	0.37ab	2.71de	4.59b
	N135	203519.26ab	5.42b	0.33bcd	3.05bcd	4.42b
W		**	**	**	**	**
N		**	**	*	*	**
W×N		**	**	*	*	**

Error bars indicate the standard errors of the means (n=3);Different lowercase letters indicate significant differences among treatments within the same year (p<0.05); Controlled irrigation (C) and flooded irrigation (F). Under each irrigation regime, four nitrogen application rates of 0(N0), 85(N85), 110(N110), and 135(N135) kg N ha^-1^ were applied. * indicates an interaction at the 0.05 level (p ≤ 0.05), ** indicates an interaction at the 0.01 level (p ≤ 0.01).

According to **Table 4** and **5**, among all treatments, the NE of rice under the C-N110 treatment was significantly higher than that under C-N0, C-N85, and C-N135, with NE values of 280,816.36 MJ·ha^-^¹ in 2021 and 224,209.41 MJ·ha^-^¹ in 2022. However, under flood irrigation, significant differences in NE were observed in 2022, with N110 and N135 showing significantly higher NE than N0 and N85. No significant difference in NE was found under flooding irrigation in 2021. At nitrogen application amounts of 0, 85, 110, and 135 kg· ha^-^¹, the average NE of rice was 192,854.47, 210,853.08, 228,861.96, and 206,380.50 MJ·ha^-^¹, respectively. The two-year average NE values across all treatments were 231,824.25 MJ·ha^-^¹ and 187,697.60 MJ·ha^-^¹. Overall, NE exhibited a clear optimum at a moderate N rate (N110), reflecting the trade-off between EO gains and the rapid increase in EI at excessive N input. Under controlled irrigation, C–N110 consistently maximized NE because it achieved high EO while limiting EI, whereas N135 increased EI without proportional EO gains, leading to lower NE. Under flood irrigation, NE differences were weaker and more year-dependent, suggesting that interannual conditions can modulate the expression of N-rate effects on NE.

The EUE of rice under controlled irrigation was significantly higher than that under flooding irrigation. Over the two-year experiment, the average EUE under controlled irrigation was 7.24 MJ·kg^-^¹, which was 11.73% higher than that under flooding irrigation, indicating that water-saving management improves energy returns mainly by reducing EI while maintaining EO. Under controlled irrigation, treatments with nitrogen amounts of 0, 85, or 110 kg· ha^-^¹had significantly higher EUE values than the treatment with 135 kg· ha^-^¹, because excessive N raises EI without proportional EO gains. The average EUE values for C-N0, C-N85, and C-N110 were 7.85, 7.42, and 7.41 MJ·kg^-^¹, respectively, which were 37.0%, 38.43%, and 28.65% higher than those of rice under flooding irrigation at the corresponding nitrogen levels.

The average SE of the flooding irrigation and controlled irrigation rice systems was 0.25 and 0.32 MJ·kg^-^¹, respectively, as shown in **Table 4** and **5**, indicating that rice production under controlled irrigation required less energy input per unit of output compared to flooding irrigation. In 2021, the SE of rice under flooding irrigation was significantly higher than that under controlled irrigation. Overall, the SE values for F-N85 and F-N110 were significantly higher than those of the other management systems.

Under different management modes, controlled irrigation rice had an average EP of 4.12 MJ·kg^-^¹, which was 28.3% higher than that under flooding irrigation. Among all treatments, C-N0, C-N85, and C-N110 exhibited significantly higher EP values than the other management systems, with average values of 4.53 MJ·kg^-^¹ in 2021 and 4.26 MJ·kg^-^¹ in 2022. These results indicated that these three treatments achieved lower EI but higher EO.

The average EPF of rice under controlled irrigation was 6.16 MJ·kg^-^¹, compared to 4.61 MJ·kg^-^¹ under flooding irrigation. Meanwhile, the EPF values for C-N0, C-N85, and C-N110 were significantly higher than those of the other treatments, with averages of 6.85 MJ·kg^-^¹ in 2021 and 6.42 and 6.41 MJ·kg^-^¹ in 2022, respectively. High EPF indicates an optimized energy input–output ratio in rice production, meaning that farmers or agribusinesses can achieve higher energy returns for the same amount of human, material, and financial inputs.

## 4 Discussion

### 4.1 Effects of irrigation modes and nitrogen fertilizer management on energy input

Total EI increased with irrigation amount (**Table 3**). Compared with flooded irrigation, controlled irrigation reduced total EI by 8.71%. Machinery and diesel contributed the largest shares of EI (**Figure 3**), consistent with Zhai et al (Zhai et al., 2021). This reflects the typical substitution of mechanization for human labor in modern rice systems (Kim et al., 2018). The effect of irrigation regime on EI was mainly driven by pumping energy requirements and the associated diesel use for field operations (Chaudhary et al., 2017). Flooded irrigation requires maintaining a standing water layer for extended periods, increasing irrigation frequency and total water use, which prolongs pump operation and raises electricity or diesel consumption (Mani and Patel, 2012). In contrast, controlled irrigation reduce irrigation volume and pumping demand, and may also improve soil physical conditions and lower traction resistance during field operations, thereby decreasing diesel consumption and overall EI (Tao et al., 2024).

Nitrogen fertilizer accounted for 79.48–86.01% of total fertilizer-related EI in rice production (**Table 3**). Excessive N application can disrupt plant physiological regulation and ultimately reduce yield (Cherati et al., 2011; Zhou et al., 2023), while also increasing production energy demand; therefore, avoiding over-application should be a key management priority. Nevertheless, many farmers still apply N at rates above crop requirements in an effort to maximize yield, and in some regions N inputs have substantially exceeded plant uptake at maximum yield, resulting in low N use efficiency (typically 20–35%). Optimizing N rates and improving N utilization while maintaining high yields is thus essential. In addition, replacing conventional urea with slow- or controlled-release fertilizers can reduce losses and enhance N efficiency (Wang et al., 2023). For example, applying blends of controlled-release fertilizers at appropriate basal rates can better match the bimodal N demand of rice, improving N use efficiency without compromising grain yield (Mi et al., 2019).

Pishgar Komleh et al (Pishgar-Komleh et al., 2011) reported that diesel and chemical fertilizers contributed 46% and 36% of total EI, respectively, in rice production in Gilan Province, Iran. Similarly, Yang et al (Yang et al., 2022) found that fertilizers, fuels, and irrigation water were the dominant EI components in Chinese rice systems, together accounting for 92.02% of total EI. These findings highlight that improved irrigation scheduling and optimized N application can simultaneously enhance N use efficiency and reduce energy demand for pumping and fertilizer use, thereby improving EUE. Consequently, mitigation efforts should prioritize EI-intensive inputs–particularly fertilizers, diesel, irrigation, and machinery–to curb total energy consumption and support sustainable rice production (Yuan and Peng, 2017).

Direct EI accounted for ~42–60% of total EI in rice production (**Figure 2**). Under the N85, N110, and N135 treatments, direct EI exceeded indirect EI, reflecting the high level of mechanization in the study area, where machinery was widely used for tillage, fertilization, irrigation, and harvesting, thereby improving operational efficiency. Nevertheless, opportunities remain to eliminate unnecessary energy use. In addition, non-renewable EI represented 78–90% of total EI (**Figure 4**), indicating a strong dependence of the rice system on non-renewable energy sources (Pishgar-Komleh et al., 2011; Pokhrel and Soni, 2017). Such dependence can increase greenhouse gas emissions and environmental burdens (Zangeneh et al., 2010). Diesel, fertilizers, and electricity were the three largest contributors to non-renewable EI. Therefore, reducing reliance on these inputs through improved management and greater adoption of renewable energy will be critical for lowering environmental impacts and enhancing ecological and energy security (Yuan and Peng, 2017).

### 4.2 Effects of irrigation modes and nitrogen fertilizer management on energy output

As illustrated in **Figure 5**, an appropriate coupling of irrigation and N management (rate and timing) can synergistically improve yield and resource-use efficiencies. Under controlled irrigation, EO increased from N0 to N110 but declined sharply at N135, becoming significantly lower than at N110 and close to N0. This suggests that excessive N under controlled irrigation reduces energy output, likely due to diminished N availability to the crop and physiological or soil-nutrient imbalances (Ladha et al., 2000). Under flooded irrigation, EO increased with N rate, although the difference between N110 and N135 was small, indicating limited marginal returns at high N input. Across N rates, EO was consistently higher under controlled than flooded irrigation. Controlled irrigation can modify soil N cycling and increase soil N availability, thereby supporting greater yield formation relative to flood irrigation (Bagheri Novair et al., 2024). Consistent with previous studies, controlled irrigation can simultaneously conserve water and sustain or increase rice yield (Johnson et al., 2024; Yang et al., 2025), improving crop growth, harvest index, and thus EO (Du et al., 2022). In our two-year experiment, C–N110 achieved the highest EO, implying greater grain and straw production. Previous work also supports these benefits: controlled irrigation increased grain yield by 5.3% compared with flooded irrigation (Tao et al., 2024) and increased crop N uptake and N use efficiency by 8.3% and 7.8%, respectively (Chen et al., 2019). Overall, water-saving irrigation tends to increase NE, and the effects of N rate on EO and NE depend strongly on irrigation regime. Therefore, integrated water–management is essential when optimizing rice production strategies.

### 4.3 Effects of irrigation modes and nitrogen fertilizer management on rice energy indicators

As shown in **Table 4** and **5**, NE, EUE, SE, EP, and EPF were highly responsive to irrigation regime. Controlled irrigation reduced water input and improved nutrient uptake, thereby increasing NE. Moderate N supply promoted rice growth by enhancing photosynthetic capacity and tillering, which increased NE (Eisapour Nakhjiri et al., 2021). In contrast, excessive N disturbed nutrient balance, increased the proportion of ineffective tillers, and reduced photosynthetic performance, ultimately lowering NE. EUE–a key indicator in agricultural energy input–output assessments—was significantly higher under C–N0, C–N85, and C–N110 than under the other treatments, indicating more efficient conversion of input energy into biomass production (Kazemi et al., 2015; Yuan and Peng, 2017). As shown in **Table 4** and **5**, different water and nitrogen management significantly impact the EUE. For example, in 2021 and 2022, the higher EUE values were observed with nitrogen levels N110. This indicates that moderate nitrogen application, combined with efficient irrigation, can significantly enhance energy utilization. This result suggests that by fine-tuning the water and nitrogen balance, energy efficiency in rice production can be improved while reducing fertilizer usage. By analyzing the data from 2021 and 2022, it was found that in the controlled irrigation method, combinations of N85 and N110 produced the higher EP. Additionally, in 2022, N85 and N110 showed favorable results in terms of EPF, suggesting that moderate nitrogen application combined with proper irrigation can improve energy production output relative to input. Therefore, in rice production, a balanced water and nitrogen management strategy can significantly increase economic returns while lowering energy consumption. In addition, a high EPF indicates that the EO of rice, primarily in the form of grain, which can be converted into food energy or other usable forms, is substantially greater relative to EI, including chemical energy from fertilizers and pesticides, as well as human and mechanical energy. This is typically reflected in higher grain yields per unit area or per unit of EI.

This study also revealed the interactive effects of nitrogen application and water management on energy efficiency. In both 2021 and 2022, significant W×N interactions were observed, suggesting that different irrigation methods combined with varying nitrogen levels have a considerable impact on energy efficiency. This finding highlights the need for an integrated approach that considers both water and nitrogen management in order to achieve optimal energy utilization and environmental sustainability in rice production. At the same time, the significant differences observed in **Table 4** and **5** can be attributed to the varying effects of water management and nitrogen fertilization levels on energy indicators over two consecutive years. In both years, the interaction between water management methods and nitrogen levels was significant, indicating that the combined effect of these factors on energy indicators was stronger than their individual influences. Notably, 2022 showed more pronounced significant differences, particularly in NE and EUE, EP, EPF. As shown in **Figure 1**, in 2022, drier conditions and insufficient effective rainfall made water a stronger limiting factor, increasing reliance on irrigation and thus amplifying differences among irrigation regimes in controlling root-zone moisture/aeration and N losses via percolation/runoff and denitrification. If rainfall was also more dispersed and temperatures were lower/more variable, stronger water-status fluctuations and constrained N assimilation would further increase the dependence of the N response on irrigation, making the W × N interaction more pronounced. As shown in **Figure 5** and **Figure 6**, under controlled irrigation, yield and EO increased to N110 (plateauing at N85–N110 in 2021) but dropped at N135, indicating over-fertilization penalties and an optimum at N110. Under flooded irrigation, yield and EO plateaued after N110, and N135 provided little benefit; moreover, higher fertilizer energy inputs at N135 led to diminishing energy returns and limited gains in NE, EUE, EP, and EPF (**Table 4**-**5**). Mechanistically, C reduces irrigation energy inputs and improves rhizosphere conditions, enhancing N uptake and allocation to productive tillers and grain; N110 matches crop demand, whereas N135 likely stimulates inefficient vegetative growth and ineffective tillers, reducing harvestable yield. Consequently, C–N110 best balances high output with minimal inputs, achieving superior net energy and energy-use efficiency (**Table 4**-**5**).

## 5 Conclusions

During the rice production process, total EI increased with irrigation amount and nitrogen fertilizer application amounts. Over 73% of the total EI in rice production originated from machinery, diesel and fertilizers. Currently, rice production faces the challenge of high dependence on non-renewable energy sources, which highlighted the resource constraints and environmental pressures associated with the current agricultural production model, indicating a need for improvement in the pursuit of sustainable farming practices. This study analyzed the energy efficiency indicators of rice fields under different water and nitrogen management methods in 2021 and 2022, finding that water and nitrogen management significantly impacted EUE, EP, and EPF. Optimal nitrogen application combined with efficient irrigation significantly enhances energy efficiency and economic returns in rice production. The study demonstrates that optimizing water and nitrogen management, especially by adjusting the nitrogen levels and irrigation methods, can significantly improve energy efficiency and economic benefits in rice production. Therefore, fine-tuned water and nitrogen management practices not only improve energy efficiency but also provide practical guidelines for sustainable agricultural practices. Under controlled irrigation conditions, total EO gradually increased under N0, N85 and N110 treatments but decreases under N135 compared to N110. After peaking at N110, total EO declines under N135. However, under flooding irrigation conditions, total EO increased with nitrogen application amount, although the increase in EO at N135 was less pronounced compared to N110. Meanwhile, results from the 2-year experiment consistently indicated that C-N110 achieved the highest levels of EO and NE among all treatments, making it well-suited for implementation in rice production regions with abundant rainfall. F-N110 demonstrated superior energy efficiency–reflected in higher EUE and EPF–compared to F-N135, due to its lower EI while maintaining similar EO. Therefore, F-N110 was suitable for promotion in rice-growing areas with favorable irrigation conditions. Among the two irrigation methods, N110 achieved the highest rice yield, indicating that a reasonable nitrogen application rate has a significant impact on the final output of rice. It should be clear that the conclusions of this study were based on the specific soil type (sandy clay loam) and rice variety (“Suijing 18”) in the cold regions of Northeast China. To develop a more universally applicable energy-saving rice production system for cold regions, future research urgently needs to establish a long-term, largescale field observation network covering diverse water–nitrogen management combinations across broader spatial and temporal scales, and to conduct in-depth analyses of the underlying crop physiological and soil microbial mechanisms. Such efforts will support theory-driven and practice-oriented advancements toward regional agricultural sustainability.

## Data Availability

The original contributions presented in the study are included in the article/supplementary material. Further inquiries can be directed to the corresponding authors.
